# Integrated Transcriptome Analysis of miRNAs and mRNAs in the Skeletal Muscle of Wuranke Sheep

**DOI:** 10.3390/genes14112034

**Published:** 2023-10-31

**Authors:** Yueying Yun, Rihan Wu, Xige He, Xia Qin, Lu Chen, Lina Sha, Xueyan Yun, Tadayuki Nishiumi, Gerelt Borjigin

**Affiliations:** 1 College of Food Science and Engineering, Inner Mongolia Agricultural University, Hohhot 010018, China; yunyueying1999@163.com (Y.Y.); hexige1212@163.com (X.H.); qinxia98@163.com (X.Q.); chenluuu0618@163.com (L.C.); huercha0505@163.com (L.S.); yunxueyan2017@imau.edu.cn (X.Y.); 2 School of Life Science and Technology, Inner Mongolia University of Science and Technology, Baotou 014010, China; 3 College of Biochemistry and Engineering, Hohhot Vocational College, Hohhot 010051, China; 15024907020@163.com; 4 Division of Life and Food Science, Graduate School of Science and Technology, Niigata University, Niigata 950-2181, Japan

**Keywords:** *Wuranke sheep*, skeletal muscle, muscle development, miRNA, mRNA

## Abstract

MicroRNAs (miRNAs) are regarded as important regulators in skeletal muscle development. To reveal the regulatory roles of miRNAs and their target mRNAs underlying the skeletal muscle development of *Wuranke sheep*, we investigated the miRNA and mRNA expression profiles in the biceps femoris of these sheep at the fetal (3 months of gestation) and 3- and 15-month-old postnatal stages. Consequently, a total of 1195 miRNAs and 24,959 genes were identified. Furthermore, 474, 461, and 54 differentially expressed miRNAs (DEMs) and 6783, 7407, and 78 differentially expressed genes (DEGs) were detected among three comparative groups. Functional analysis demonstrated that the target mRNAs of the DEMs were enriched in multiple pathways related to muscle development. Moreover, the interactions among several predicted miRNA–mRNA pairs (*oar-miR-133*-*HDAC1*, *oar-miR-1185-5p*-*MYH1*/*HADHA*/*OXCT1*, and *PC-5p-3703_578*-*INSR*/*ACTG1*) that potentially affect skeletal muscle development were verified using dual-luciferase reporter assays. In this study, we identified the miRNA and mRNA differences in the skeletal muscle of *Wuranke sheep* at different developmental stages and revealed that a series of candidate miRNA–mRNA pairs may act as modulators of muscle development. These results will contribute to future studies on the function of miRNAs and their target mRNAs during skeletal muscle development in *Wuranke sheep*.

## 1. Introduction

Skeletal muscle, the largest tissue by body mass, is closely related to the meat production and quality of livestock. The development of skeletal muscle is a complex and multistep biological process that has distinct embryonic and postnatal phases. The embryonic period, the key period of myogenesis, includes the formation, proliferation, and differentiation of myoblasts, the fusion of myotubes, and the maturation of myofibers. The number of muscle fibers is determined during the embryonic period and remains constant after birth, while postnatal muscle development is mainly manifested by an increase in muscle fiber size [1]. These processes are precisely regulated by a complex molecular regulatory network that is composed of a number of genetic factors [2,3].

MicroRNAs (miRNAs), a class of endogenous non-coding RNAs ranging in length from 18 to 25 nucleotides, act as post-transcriptional regulators of target gene expression by promoting mRNA decay or translational repression; they, thus, mediate a variety of biological processes [4,5]. It is known that cell proliferation and differentiation are crucial for the myogenic program, and increasing evidence suggests that many miRNAs play regulatory roles in balancing these two processes by regulating the expression of specific genes, including both the muscle-specific and non-muscle-specific miRNAs reviewed by Xu et al. [6]. Additionally, other studies showed that some miRNAs are also involved in the processes of skeletal muscle regeneration [7], muscle-fiber-type transformation [8], and energy metabolism [9].

Recently, several studies have analyzed miRNA profiles in sheep skeletal muscle using a miRNA sequencing approach. Zhao et al. [10] analyzed the longissimus dorsi muscle (LDM) at four developmental stages of Duolang sheep and obtained a total of 2396 miRNAs, including 1920 novel miRNAs. Further research found that some of these were differentially expressed among the four stages; additionally, *miR-192* was confirmed to regulate the proliferation and differentiation of satellite cells in the skeletal muscle of sheep by targeting retinoblastoma 1 (*RB1*), a negative regulator of the cell cycle, which is involved in myogenesis. Liu et al. [11] identified 1086 known and 40 novel miRNAs in embryonic and adult Kazak sheep, 345 of which were differentially expressed. It was shown that these differentially expressed miRNAs (DEMs) were enriched in multiple signaling pathways related to muscle development. Among them, several were identified as regulators of sheep skeletal muscle cell proliferation and differentiation, such as *miR-27b* [12], *miR-378* [13], *miR-181a* [14], and *miR-22* [15]. Hu et al. [16] studied the expression profiles of miRNAs and their potential roles in the muscle of Chinese Merino sheep at three embryonic stages, detecting 4752 miRNAs, including 2275 novel ones. The expression of *miR-410-5p* was reduced during the embryonic period and restrained the proliferation of embryonic myoblasts by interacting with TEA domain transcription factor 1 (*TEAD1*), which activates several muscle-specific genes and regulates myoblast proliferation and differentiation. These studies were limited to the regulation of miRNAs relating to the proliferation and differentiation of sheep skeletal muscle cells, and, compared with the detected sheep miRNAs, most of their functions remain unclear. Thus, the identification of more potential muscle-associated miRNA–mRNA pairs is required in order to reveal such pairs’ underlying regulatory mechanisms.

The *Wuranke sheep* is an ancient Mongolian breed mainly raised in the northern border area of Abaga, Xilingol, Inner Mongolia, China (E 113°28′–116°11′, N 43°05′–45°26′), a region characterized by severe cold, drought, and wind. Through long-term natural selection and artificial breeding, these sheep demonstrate strong adaptability to the local alpine pastoral ecological environment, a high coarse feeding tolerance, a rapid growth rate, and good meat production performance under natural grazing conditions. Furthermore, they are very popular with consumers because of their delicate flesh and good flavor. Previous studies measured some indicators that reflect the meat performance of *Wuranke sheep*, such as body weight, body size, and carcass traits, but the molecular regulatory mechanisms involved in skeletal muscle development in this breed remain unclear. To better understand the functions of miRNAs and their target mRNAs that underlie skeletal muscle development in *Wuranke sheep*, we employed high-throughput sequencing to analyze and compare the expression profiles of miRNA and mRNA in the skeletal muscle of natural grazing *Wuranke sheep* at three developmental stages. The DEMs between any two stages and their candidate target mRNAs were identified and used for functional enrichment in order to reveal the potential miRNA–mRNA pairs involved in the process of muscle development. Finally, we constructed three miRNA–mRNA interaction networks, and the predicted targets of three miRNAs were validated via dual luciferase reporter assays. The results provided valuable information about the regulatory mechanisms of skeletal muscle development and basic data on the breeding and selection of *Wuranke sheep*.

## 2. Materials and Methods

### 2.1. Sample Collection and RNA Extraction

*Wuranke sheep* used in this study were collected from the “The original Breeding Farm of *Wuranke Sheep*” in Abaga Banner, Inner Mongolia, China. All the animals were raised under natural grazing conditions. Pregnant ewes and 3- and 15-month-old male sheep were randomly selected. Male fetuses were from a single birth and were collected at 3 months of gestation via cesarean section. Biceps femoris samples were collected from three male fetuses, three 3-month-old male sheep, and three 15-month-old male sheep, which were as close to the median average body weight of their groups as possible. The collected samples were immediately placed in liquid nitrogen and then stored at −80 °C. miRNeasy Mini Kit (Qiagen, Hilden, Germany) was used to extract the total RNA from each sample. The Agilent 2100 Bioanalyzer and RNA 6000 Nano LabChip Kit (Agilent Technologies, Santa Clara, CA, USA) were used to detect the total RNA quantity and purity, and an RNA integrity number of >7.0 was set as the selection criterion.

### 2.2. Small RNA Sequencing and Data Analysis

Small RNA libraries were constructed with approximately 1 μg of total RNA using the TruSeq Small RNA Sample Prep Kits (Illumina, San Diego, CA, USA). Then, the libraries were used for single-end (1 × 50 bp) deep sequencing using an Illumina HiSeq 2500 platform. The raw reads were submitted to ACGT101-miR v4.2 (LC Sciences, Houston, TX, USA) to remove adapter dimers, junk, low complexity, common RNA families (rRNA, tRNA, snRNA, and snoRNA), and repeats. Next, unique sequences ranging in length from 18 to 26 nucleotides were aligned to all mature mammalian miRNAs and their precursor sequences in miRBase to identify known miRNAs using BLAST search. In addition, the remaining unmapped sequences that we were able to match to the sheep reference genome (*Ovis aries* v3.1) were used to predict novel miRNAs using miRDeep2 [17]. Meanwhile, the expression level of each miRNA was normalized using the method described by Li et al. [18], and differential miRNA expressions were analyzed using *t*-tests. miRNAs with |log2 (fold-change)| ≥ 1 and *p* < 0.05 were deemed to be differentially expressed between any two developmental stages.

### 2.3. mRNA Sequencing and Data Analysis

mRNA libraries were constructed with approximately 10 μg of total RNA using the mRNA-Seq Sample Preparation Kit (Illumina, San Diego, CA, USA). Then, the libraries were used for paired-end reads (2 × 150 bp) deep sequencing using an Illumina HiSeq 4000 platform. The raw data were subjected to Cutadapt [19] to remove adaptor sequences, primers, and low-quality sequences in order to obtain clean reads. The HISAT v2.0.4 package (https://daehwankimlab.github.io/hisat2/, accessed on 21 August 2020) [20] was used to align clean reads to the sheep reference genome (*Ovis aries* v3.1), and StringTie v1.3.4 (http://ccb.jhu.edu/software/stringtie/, accessed on 21 August 2020) [21] was used to assemble the mapped reads and evaluate the expression levels for mRNAs by calculating fragments per kilobase of exon model per million mapped reads (FPKM). Finally, differentially expressed genes (DEGs) with |log2 (fold-change)| ≥ 1 and *p* < 0.05 as thresholds were identified using the R package Ballgown [22].

### 2.4. Real-Time Quantitative Polymerase Chain Reaction (RT-qPCR) Analysis

Total RNA was reverse-transcribed using the RevertAid First Strand cDNA Synthesis Kit (Thermo, Waltham, MA, USA), following the manufacturer’s instructions, and using stem-loop primers for miRNA and random primers for mRNA. RT-qPCR was performed using the ABI StepOnePlus Real-Time PCR System (Applied Biosystems) with THUNDERBIRD SYBR qPCR Mix (Toyobo, Osaka, Japan). *U6* and *GAPDH* were used as internal reference genes in normalizing miRNA and mRNA expression, respectively. The 2^−ΔΔCt^ method was employed to measure the relative gene expression [23]. The results are presented as mean ± standard error (SE) of triplicate for each sample. The sequences of the primers are listed in Tables S1 and S2.

### 2.5. Target Gene Prediction and Functional Analysis

To predict target mRNAs of DEMs, we used TargetScan 50 and miRanda 3.3a algorithms to identify miRNA binding sites. The predicted targets obtained using both algorithms were calculated. Then, the results were combined with the DEG data. ACGT101-CORR v1.1 was used to analyze differentially expressed miRNA–mRNA correlations. Only target mRNAs with inverse relationships with corresponding miRNAs were identified as candidate target mRNAs. To predict the potential function of candidate target mRNAs, we aligned them against the Gene Ontology (GO) [24] and Kyoto Encyclopedia of Genes and Genomes (KEGG) [25] databases. The GO functional annotation is categorized into three ontologies: biological process (BP), cellular components (CC), and molecular functions (MF). GO function and KEGG pathway enrichment analysis were performed using the Goseq v1.18.0 (https://bioconductor.org/packages/3.0/bioc/html/goseq.html, accessed on 30 August 2020) [26] and KOBAS v2.0 (http://bioinfo.org/kobas/, accessed on 30 August 2020) packages [27], respectively. A *p*-value of < 0.05 was defined as statistically significant. Then, the regulatory networks of miRNA–mRNA interactions related to muscle development in all pairwise comparisons were visualized using Cytoscape v3.9.0 (http://www.cytoscape.org/, accessed on 10 September 2021).

### 2.6. Dual-Luciferase Reporter Assays

miRNA mimics and the negative control (NC) mimics were synthesized by GenePharama (Shanghai, China). Wild-type (WT) and mutant (MUT) luciferase reporter vectors were constructed by cloning predicted sequences or the corresponding mutated sequences for the 3′UTR of individual genes into psiCHECK-2 vectors (Promega, Madison, WI, USA). Before transfection, 293T cells were seeded in 96-well plates (3 × 10^4^ cells/well) and incubated at 37 °C. At 70% confluency, the cells were cotransfected using the WT or MUT constructs along with miRNA mimics or NC mimics using LipoFiterTM Liposomal Transfection Reagent (Hanbio, Shanghai, China). After 48 h of transfection, the firefly and Renilla luciferase activities were detected with an Infinite M1000 PRO multimode reader (Tecan, Mannedorf, Switzerland) using the dual-luciferase assay system (Promega, Madison, WI, USA).

### 2.7. Statistical Analysis

The RNA-seq and RT-qPCR data were analyzed as described above. The Renilla luciferase activity data were analyzed using Student’s *t*-test and presented as mean ± standard error (SE). A *p*-value < 0.05 was deemed as statistically significant. The principal component analyses (PCA) of the miRNA and mRNA expression profiles were performed using the OmicStudio tools at https://www.omicstudio.cn/tool, accessed on 21 August 2020.

## 3. Results

### 3.1. Overview of Small RNA and mRNA Sequencing Data

**Figure 1:**
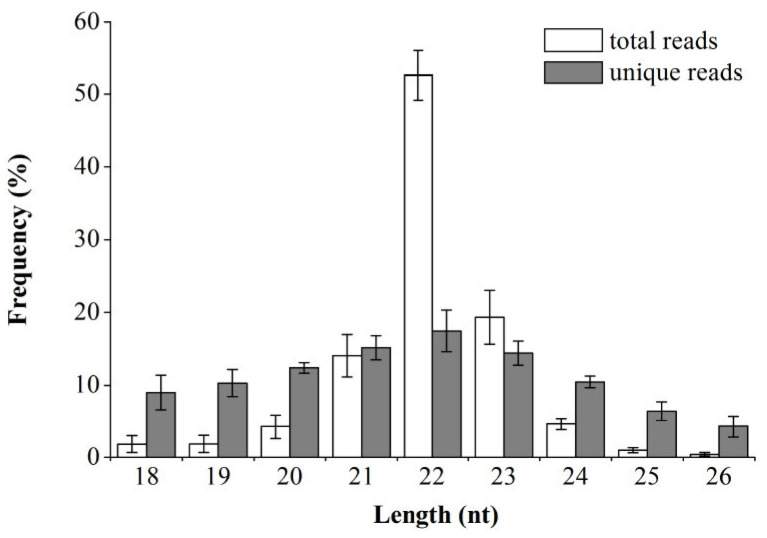
Sequence length distribution of total and unique valid reads. Data are presented as mean ± SD from nine small RNA libraries.

During the small RNA sequencing, a total of 109,558,270 raw reads were generated. After filtering and size selection were carried out, 91,104,586 valid reads were obtained (Table S3). The majority of the small RNA reads were 21–23 nt in size, with a length of 22 nt being the most common (Figure 1), which was in line with the characteristics of classic dicer-processed products and the mature miRNA length distribution [28]. The valid reads were aligned to the reference sequence. A total of 1195 miRNAs were identified, including 988 known miRNAs and 207 novel miRNAs. Based on normalized counts, high expression levels were observed in the known miRNAs, with *oar-miR-133* being the most abundant miRNA (Table S4). The novel miRNAs had much lower expression levels than the known miRNAs, with only one miRNA (*PC-3p-282_33957*) having average normalized counts greater than 1000 at any of the studied developmental stages (Table S5). During mRNA sequencing, a total of 484,263,900 raw reads were generated, and more than 98% of them were found to be valid reads after the low-quality bases were filtered out. On average, about 91.58% of the valid reads were mapped to the sheep reference genome from each library (Table S6), suggesting the high quality of the sequencing results.

**Figure 2:**
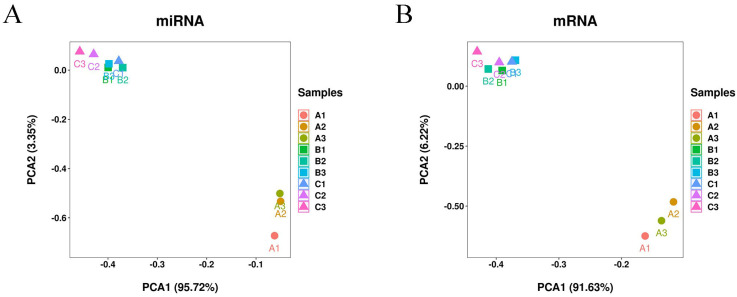
Principal component analysis (PCA) for miRNA (**A**) and mRNA (**B**) expression profiles. Fetal: A1, A2, and A3; 3-month-old: B1, B2, and B3; 15-month-old: C1, C2, and C3.

Then, the mapped reads were assembled into 24,959 genes. The PCA analysis of the miRNA (Figure 2A) and mRNA (Figure 2B) expression profiles showed that the triplicate samples for each group were clustered together, and that the 3- and 15-month-old samples were more similar to each other than and robustly separated from the fetal samples, suggesting that there are great differences between fetal sheep and 3- and 15-month-old sheep.

### 3.2. Differential Expression Analysis of miRNAs and mRNAs

**Figure 3:**
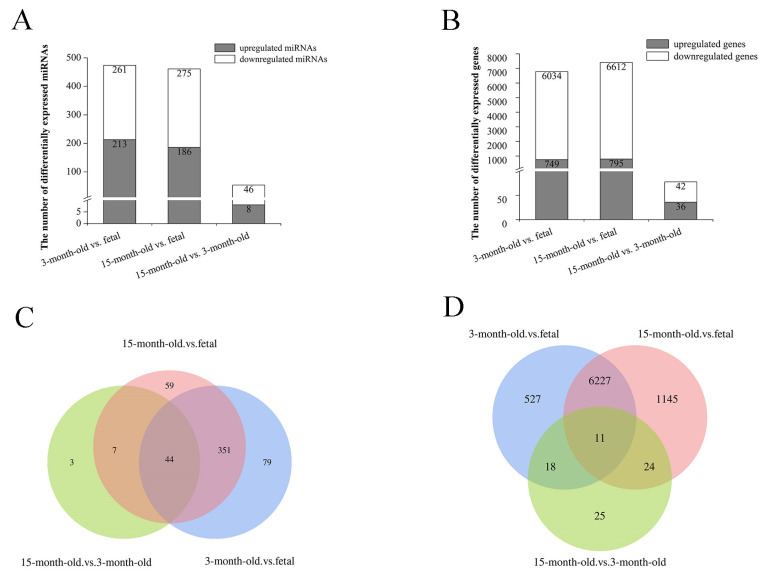
Analysis of differentially expressed miRNAs (DEMs) and differentially expressed genes (DEGs) in the 3-month-old vs. fetal, 15-month-old vs. fetal, and 15-month-old vs. 3-month-old comparisons. (**A**) Numbers of upregulated and downregulated DEMs in each pairwise comparison. (**B**) Numbers of upregulated and downregulated DEGs in each pairwise comparison. (**C**) Venn diagram of DEMs in each pairwise comparison. (**D**) Venn diagram of DEGs in each pairwise comparison.

We compared the miRNA and mRNA expression levels of each stage with the others (3-month-old vs. fetal, 15-month-old vs. fetal, and 15-month-old vs. 3-month-old). As a result, 474 DEMs (213 upregulated and 261 downregulated) were obtained in the 3-month-old vs. fetal comparison, including 23 novel miRNAs. In the 15-month-old vs. fetal comparison, 461 DEMs (186 upregulated and 275 downregulated) were identified, including 17 novel miRNAs. In the 15-month-old vs. 3-month-old comparison, 54 miRNAs (8 upregulated and 46 downregulated) were differentially expressed, but no novel miRNAs were detected (Figure 3A). We also obtained 6783 DEGs (749 upregulated and 6034 downregulated), 7407 DEGs (795 upregulated and 6612 downregulated), and 78 DEGs (36 upregulated and 42 downregulated) in the 3-month-old vs. fetal, 15-month-old vs. fetal, and 15-month-old vs. 3-month-old comparisons, respectively (Figure 3B). Most of these DEMs and DEGs were found at the intersection of the 3-month-old vs. fetal and 15-month-old vs. fetal comparisons, with the lowest number of these being found in the 15-month-old vs. 3-month-old comparison (Figure 3C,D). These findings indicated there were distinct miRNA and mRNA expression patterns between the fetal and the two postnatal (3- and 15-month-old) samples, which supports the results of the PCA analysis mentioned above.

### 3.3. Validation of RNA-seq Data Using RT-qPCR

**Figure 4:**
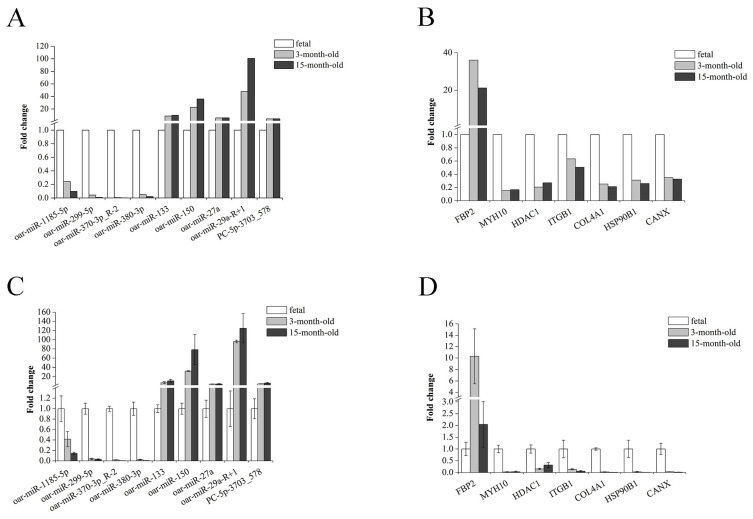
Validation of RNA-seq data using real-time quantitative polymerase chain reaction (RT-qPCR). (**A**) RNA-seq data for DEMs. (**B**) RNA-seq data for DEGs. (**C**) RT-qPCR analysis of DEMs. (**D**) RT-qPCR analysis of DEGs.

To validate the sequencing data, nine DEMs (*oar-miR-1185-5p*, *oar-miR-299-5p*, *oar-miR-370-3p_R-2*, *oar-miR-380-3p*, *oar-miR-133*, *oar-miR-150*, *oar-miR-27a*, *oar-miR-29a_R+1*, and *PC-5p-3703_578*) and seven DEGs (*FBP2*, *MYH10*, *HDAC1*, *ITGB1*, *COL4A1*, *HSP90B1*, and *CANX*) were randomly selected for examination of their expression levels using RT-qPCR (Figure 4). The expression trends observed using RT-qPCR were consistent with those based on the sequencing data, indicating that the sequencing results were reliable.

### 3.4. Integrated miRNA–mRNA Interaction Analysis

**Figure 5:**
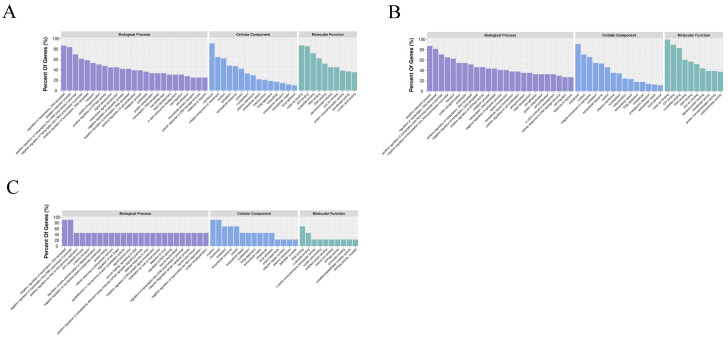
Gene Ontology (GO) enrichment analysis of candidate target mRNAs in 3-month-old vs. fetal (**A**), 15-month-old vs. fetal (**B**), and 15-month-old vs. 3-month-old (**C**) comparisons.

**Figure 6:**
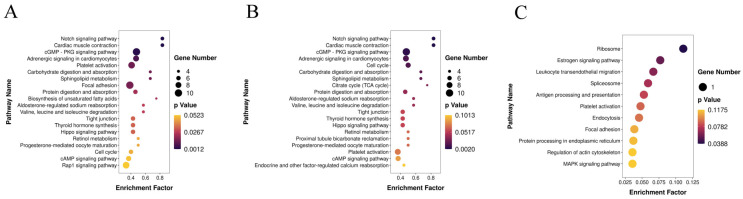
Kyoto Encyclopedia of Genes and Genomes (KEGG) pathway enrichment analysis of candidate target mRNAs in 3-month-old vs. fetal (**A**), 15-month-old vs. fetal (**B**), and 15-month-old vs. 3-month-old (**C**) comparisons.

As described in the “Materials and Methods” Section, we obtained the candidate target mRNAs of all the DEMs for further analysis. The enriched GO functions for the candidate target mRNAs in the 3-month-old vs. fetal comparison showed a high similarity with those in the 15-month-old vs. fetal comparison and were mainly enriched in the “regulation of transcription, DNA–templated” and “oxidation–reduction process” terms in the BP ontology, the “membrane” and “integral component of membrane” terms in the CC ontology, and the “metal ion binding” and “ATP binding” terms in the MF ontology (Figure 5A,B). Alternatively, in the 15-month-old vs. 3-month-old comparison, the candidate target mRNAs were primarily enriched in the “negative regulation of transcription, DNA-templated” and “negative regulation of transcription from RNA polymerase II promoter” terms in the BP ontology, the “nucleus” and “membrane” terms in the CC ontology, and the “RNA binding” and “nucleotide binding” terms in the MF ontology (Figure 5C). Some muscle-related GO terms were also enriched, including the “cell proliferation”, “cell migration”, “cell adhesion”, “myotube differentiation”, and “canonical Wnt signaling pathway” terms. The KEGG pathway analysis revealed that the candidate target mRNAs of the DEMs were significantly enriched in 18, 11, and 1 pathways in the 3-month-old vs. fetal (Figure 6A), 15-month-old vs. fetal (Figure 6B), and 15-month-old vs. 3-month-old comparisons (Figure 6C), respectively (*p* < 0.05). Notably, the “PI3K–Akt signaling pathway” was enriched by the largest number of candidate target mRNAs in both the 3-month-old vs. fetal and 15-month-old vs. fetal comparisons, although the enrichment was not significant (*p* > 0.05). Most of these pathways were associated with muscle growth and development, including the “Notch signaling pathway”, “PI3K-Akt signaling pathway”, and “Hippo signaling pathway”, as well as the “focal adhesion”; “tight adhesion”; “cell cycle”; “citrate cycle”; “biosynthesis of unsaturated fatty acids”; “sphingolipid metabolism”; “retinol metabolism”; “valine, leucine and isoleucine degradation”; “thyroid hormone synthesis”; and “ribosome” pathways.

**Figure 7:**
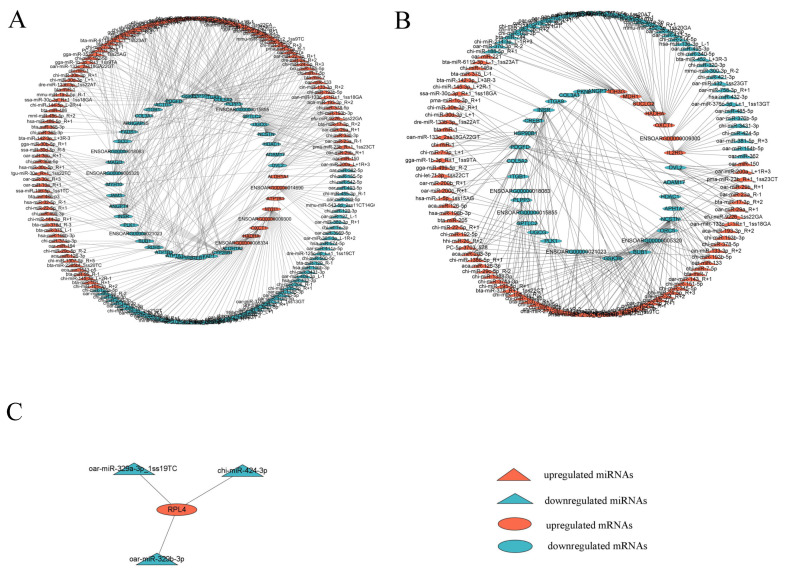
Regulatory networks of miRNA–mRNA pairs related to muscle development in 3-month-old vs. fetal (**A**), 15-month-old vs. fetal (**B**), and 15-month-old vs. 3-month-old (**C**) comparisons. Red triangles represent upregulated miRNAs, blue triangles represent downregulated miRNAs, red ellipses represent upregulated mRNAs, and blue ellipses represent downregulated mRNAs.

Then, we constructed a miRNA–mRNA interaction network for each comparison using DEMs with average normalized counts greater than 100 and their candidate target mRNAs that were enriched in KEGG pathways associated with muscle development. As a result, a total of 401 differentially expressed miRNA–mRNA pairs (163 miRNAs and 45 mRNAs), 267 differentially expressed miRNA–mRNA pairs (123 miRNAs and 33 mRNAs), and 3 differentially expressed miRNA–mRNA pairs (3 miRNAs and 1 mRNA) with negative regulatory relationships were detected in the 3-month-old vs. fetal (Figure 7A), 15-month-old vs. fetal (Figure 7B), and 15-month-old vs. 3-month-old (Figure 7C) comparisons, respectively.

### 3.5. Validation of miRNA–mRNA Interaction Using Dual-Luciferase Reporter Assays

**Figure 8:**
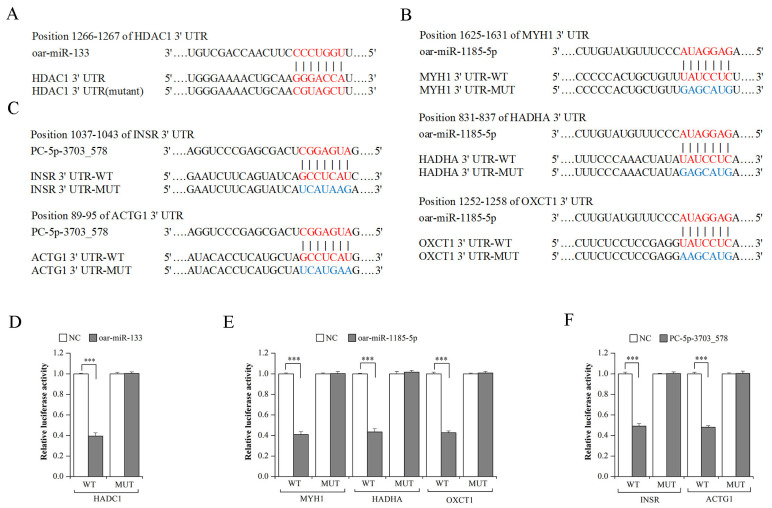
Prediction and validation of *oar-miR-133*, *oar-miR-1185-5p*, and *PC-5p-3703_578* target mRNAs. (**A**) The predicted binding sites of *oar-miR-133* in the 3′UTR of *HADC1*. (**B**) The predicted binding sites of *oar-miR-1185-5p* in the 3′UTR of *MYH1*, *HADHA*, and *OXCT1*. (**C**) The predicted binding sites of *PC-5p-3703_578* in the 3′UTR of *INSR* and *ACTG1*. (**D**) The luciferase reporter assays were performed on 293T cells by cotransfecting the *HADC1* 3′UTR-WT or 3′UTR-MUT psiCHECK-2 vector along with either *oar-miR-133* mimics or NC mimics. (**E**) The luciferase reporter assays were performed on 293T cells by cotransfecting the *MYH1*, *HADHA* or *OXCT1* 3′UTR-WT or 3′UTR-MUT psiCHECK-2 vector along with either *oar-miR-1185-5p* mimics or NC mimics. (**F**) The luciferase reporter assays were performed on 293T cells by cotransfecting the *INSR* or *ACTG1* 3′UTR-WT or 3′UTR-MUT psiCHECK-2 vector along with either *PC-5p-3703_578* mimics or NC mimics. *** *p* < 0.001.

To verify the interactions between the miRNAs and mRNAs involved in these networks, six miRNA–mRNA interactions were analyzed using dual-luciferase reporter assays. The results showed that the luciferase activity in cells cotransfected with *oar-miR-133* mimics and the *HDAC1* 3′UTR-WT vector; *oar-miR-1185-5p* mimics and the *MYH1*, *HADHA,* or *OXCT1* 3′UTR-WT vector; and *PC-5p-3703_578* mimics and the *INSR* or *ACTG1* 3′UTR-WT vector was significantly reduced compared with those transfected with NC mimics (*p* < 0.001), whereas there was no effect on the corresponding mutant reporter activity (Figure 8). These data suggest that *HDAC1* is a direct target of *oar-miR-133*; *MYH1*, *HADHA*, and *OXCT1* are direct targets of *oar-miR-1185-5p*; and *INSR* and *ACTG1* are direct targets of *PC-5p-3703_578*.

## 4. Discussion

miRNAs are recognized as important regulators of skeletal muscle development because of their modulation of gene expression. Thus, it is of great significance to study their roles in skeletal muscle development. Muscle fibers are formed prenatally in sheep, especially during mid-gestation [29]. In contrast, their postnatal muscle growth largely relies on muscle fiber hypertrophy [30]. The growth rate of sheep is extremely rapid between birth and six months of age. After 1–1.5 years of growth, their skeletal muscle fibers are close to their mature size [31]. Therefore, high-throughput sequencing technology was used to characterize the miRNA and mRNA expression profiles of the biceps femoris of *Wuranke sheep* at the fetal (3 months of gestation) and 3- and 15-month-old postnatal stages in this study.

We identified 1195 miRNAs (988 known and 207 novel miRNAs) in the small RNA libraries. A total of 543 miRNAs were significantly differentially expressed among the three stages, with most of these miRNAs being found both in the 3-month-old vs. fetal and 15-month-old vs. fetal comparisons, including some miRNAs that are highly expressed in skeletal muscle, such as *oar-miR-133*, *bta-miR-1*, *chi-miR-206_R-1*, *oar-miR-127_L-1*, and *chi-miR-378-3p*. The muscle-specific miRNAs *miR-1*, *miR-133*, and *miR-206* were extensively studied for their regulatory roles in multiple aspects of skeletal muscle development [32]. It was shown in previous studies that the main function of *miR-1/miR-206* is to promote myogenic differentiation [33], while the main function of *miR-133* is to promote myogenic proliferation [34]. Furthermore, it was reported that *miR-133a* and *miR-206* can regulate the muscle-fiber-type transition in mice [35,36]. A local injection of double-stranded *miR-1*, *miR-133*, and *miR-206* in a rat skeletal muscle injury model was found to contribute to muscle regeneration [37]. Further, *miR-127* was found to be a differentially expressed miRNA in the prenatal and postnatal skeletal muscle of sheep [11] and pigs [38]. Studies with C2C12 cells revealed that *miR-127* regulates myoblast proliferation and differentiation, and its experimentally confirmed target genes include vesicle-associated membrane protein 2 (*VAMP2*) [39], sphingosine-1-phosphate receptor 3 (*S1PR3*) [40], lysine methyltransferase 5A (*KMT5A*) [41], and septin 7 (*SEPT7*) [42]. *miR-378* was reported to facilitate C2C12 cell differentiation by targeting myogenic repressor (*MYOR*) [43] and bone morphogenetic protein 4 (*BMP4*) [44] as well as to promote the differentiation of the bovine skeletal-muscle-derived satellite cell by targeting DNA polymerase alpha subunit B (*POLA2*) [45]. In addition, *miR-378* was verified to promote myoblast proliferation in sheep [13].

In the present study, the expression levels of *oar-miR-133*, *bta-miR-1*, and *chi-miR-378-3p* were significantly higher in the muscle of 3- and 15-month-old sheep than those of fetal sheep, while the expression levels of *chi-miR-206_R-1* and *oar-miR-127_L-1* showed the opposite pattern. These results suggested that different types of miRNA may play crucial regulatory roles in different phases of sheep skeletal muscle development. Therefore, the study of miRNA expression profiles in the skeletal muscle of *Wuranke sheep* at different developmental stages is helpful for us to discover potential miRNAs and their regulatory mechanisms related to skeletal muscle development.

By regulating their target genes’ expression, miRNAs exert their functions at the post-transcriptional level. To perform miRNA–mRNA integrated analysis, we simultaneously characterized the mRNA expression profiles at the three developmental stages. A total of 7978 DEGs were identified through pairwise comparisons of the three stages; the majority of them were differentially expressed in the 3- and 15-month-old muscle samples compared to in the fetal muscle samples. Some of these DEGs are known for their crucial roles in regulating skeletal muscle development, such as myogenic factor 5 (*MYF5*) [46], myogenin (*MYOG*) [47], myocyte enhancer factor 2C (*MEF2C*) [48], and paired box 7 (*PAX7*) [49]. Furthermore, in order to reveal the potential miRNAs that regulate muscle development, the candidate target mRNAs of the DEMs were determined using integrated analysis.

The functional enrichment analysis revealed that the candidate target mRNAs were enriched in 13 pathways that are related to muscle growth and development. Among them, the “Notch signaling pathway” [50,51], “PI3K–Akt signaling pathway” [52], and “Hippo signaling pathway” [53,54], as well as the “focal adhesion” [55], “tight adhesion” [56], and “cell cycle” [57] pathways, were confirmed to regulate muscle proliferation and differentiation. Metabolic pathways, including the “citrate cycle” [58]; “biosynthesis of unsaturated fatty acids” [59]; “sphingolipid metabolism” [60]; “retinol metabolism” [61,62]; “valine, leucine and isoleucine degradation” [63]; and “thyroid hormone synthesis” [35,64] pathways, are closely associated with muscle development. Notably, these pathways were not enriched in the 15-month-old vs. 3-month-old comparison. As mentioned above, there were great differences in the mechanisms of skeletal muscle development between the fetal and postnatal period, which suggests that the DEMs and their target mRNAs that were enriched in these pathways play more important roles in the early stages of skeletal muscle development. In the 15-month-old vs. 3-month-old comparison, the “ribosome” pathway was the only significantly enriched pathway (*p* < 0.05). It is known that ribosomes serve as macromolecular machines for protein synthesis, with such machines being responsible for muscle growth [65,66]. Therefore, the miRNA–mRNA pairs involved in this pathway may exert roles in the regulation of protein synthesis rates, thus impacting skeletal muscle development.

Previous studies revealed that miRNAs with lower abundances have no discernible regulatory effects on their target genes [67,68]. Therefore, we constructed interaction networks for each comparison after filtering out the lowly expressed DEMs (average normalized counts < 100). In the interaction networks, each miRNA was shown to interact with one or more mRNAs, and vice versa, highlighting the complex regulatory roles of miRNAs.

Among the DEMs found in the networks, *oar-miR-133* was the most abundantly expressed miRNA; its expression was significantly increased after birth. We further confirmed that histone deacetylase 1 (*HDAC1*) was the target of *oar-miR-133*, according to a dual-luciferase reporter assay. As a known muscle-specific miRNA, *miR-133* was reported as a regulator in muscle cell proliferation and differentiation by targeting the serum response factor (*SRF*) [34], insulin-like growth factor-1 receptor (*IGF-1R*) [69], uncoupling protein 2 (*UCP2*) [70], forkhead transcriptional factor 2 (*FOXL2*) [71], etc. However, no studies to date suggest that *miR-133* and *HDAC1* interact. *HDAC1* plays a inhibiting role in the skeletal muscle myogenesis by suppressing the transcriptional activities of myoblast determination protein (*MYOD*), a key regulator of muscle differentiation [72]. Therefore, we hypothesized that increases in *oar-miR-133* expression may promote sheep skeletal muscle cell differentiation by downregulating *HDAC1*; if confirmed, this would extend our understanding of how *miR-133* regulates skeletal muscle development.

Among the mRNAs whose expression levels were increased in the networks, myosin heavy chain 1 (*MYH1*), hydroxyacyl-CoA dehydrogenase/3-ketoacyl-CoA thiolase/enoyl-CoA hydratase (trifunctional protein), alpha subunit (*HADHA*), and 3-oxoacid CoA-transferase 1 (*OXCT1*) were the three genes targeted by the highest number of miRNAs. *MYH1*, a highly expressed gene in MyHC-IIx fibers (fast-twitch fibers), is crucial for skeletal muscle development and can be used as a myoblast differentiation marker gene [73,74]. The upregulation of *MYH1* expression indicates that the proportion of MyHC-IIx fibers increased. Previous studies showed that muscle fiber composition is correlated with muscle fiber diameter and that the transformation of slow-twitch fibers into fast-twitch fibers can induce an increase in muscle mass [75,76]. Both *HADHA* and *OXCT1* are genes related to metabolism. *HADHA* encodes the subunit of the mitochondrial trifunctional protein, a key enzyme for the β-oxidation of fatty acid [77]. *OXCT1* encodes succinyl-CoA:3-oxoacid CoA transferase (SCOT), which is a key enzyme for ketolysis [78]. In skeletal muscle, mitochondrial fatty acid β-oxidation and ketolysis represent aerobic energy sources [79,80]. Zheng et al. [81] detected the expression of *OXCT1* and found it is associated with chicken skeletal muscle hypertrophy, while Komatsu et al. [82] demonstrated that *HADHA* expression in skeletal muscle is associated with the growth rate of pigs. In our research, *oar-miR-1185-5p* was found to be significantly downregulated in all three comparison groups and to have interacted with the abovementioned three mRNAs. *MYH1* was found to be involved in the “tight junction” pathway, *OXCT1* in the “valine, leucine and isoleucine degradation” pathway, and *HADHA* in both the “valine, leucine and isoleucine degradation” and the “biosynthesis of unsaturated fatty acids” pathways. These results implied that *oar-miR-1185-5p* may play a role in sheep muscle development through the related cellular process and metabolism pathways. To the best of our knowledge, no research has yet been reported on *miR-1185* roles during skeletal muscle development. Therefore, the results obtained from this research may provide a new perspective for understanding the regulatory mechanisms of skeletal muscle.

Further, we found that *PC-5p-3703_578* was the only novel miRNA that was involved in the networks and that interacted with insulin receptor (*INSR*) and actin gamma 1 (*ACTG1*). It is widely known that insulin is an important anabolic hormone in skeletal muscle [83]. *INSR* is an insulin receptor with tyrosine kinase activity, which acts as a molecular switch in the insulin signal transduction pathway [84]. Following the binding of insulin to *INSR*, the PI3K/AKT and MAPK/ERK signaling pathways, which regulate muscle development, are initiated, which, in turn, leads to muscle hypertrophy [85,86]. Furthermore, a recent study using C2C12 cells showed that the knockdown of *INSR* induced cell cycle arrest at G1/G0 and inhibited cell proliferation [87]. The *ACTG1*-encoded protein (cytoplasmic γ-1-actin), an actin isoform, is involved in cytoskeleton maintenance [88]. Previous studies revealed that *ACTG1* has a regulating effect in the myogenic cell migration, which is necessary for skeletal muscle formation [1,89]. Other studies found that knocking out *ACTG1* leads to growth delay and skeletal myopathy in mice [90,91]. From these results, we can speculate that *PC-5p-3703_578* may be a potential negative regulator of skeletal muscle development.

Altogether, the results of the present study provide reference data that will be of great use for investigating the regulatory mechanisms of skeletal muscle development in *Wuranke sheep*.

## 5. Conclusions

In this study, it is revealed that a series of candidate miRNA–mRNA pairs may act as modulators of muscle development. This study’s findings provide a theoretical basis for a deeper understanding of the functions of the miRNAs and their candidate target mRNAs that underlie skeletal muscle development in sheep.

## Data Availability

The sequence data of the present study were submitted to SRA with bioproject numbers PRJNA1009824 and PRJNA1009886.

## Supplementary Materials

The following supporting information can be downloaded at https://www.mdpi.com/article/10.3390/genes14112034/s1. Table S1: Primer sequences of miRNAs for RT-qPCR used in this study; Table S2: Primer sequences of mRNAs for RT-qPCR used in this study; Table S3: Summary of small RNA sequencing data; Table S4: Expression levels of top 10 highly expressed known miRNAs; Table S5: Expression levels of top 10 highly expressed novel miRNAs; Table S6: Summary of mRNA sequencing data.
