# Supplementary material for: Integrated Transcriptome Analysis of miRNAs and mRNAs in the Skeletal Muscle of Wuranke Sheep
Source: Genes (Basel). 2023 Oct 31;14(11):2034. doi: 10.3390/genes14112034 (PMC10671749; doi:10.3390/genes14112034)
Supplement: Supplementary file 1 [file genes-14-02034-s001.zip › Supplementary materials/Table S3.pdf]

Table S3. Summary of small RNA sequencing data

Sample	Raw reads	Valid reads	Q20 (%)	Q30 (%)	GC content (%)
A1	12472153	9700885	97.02	89.83	50.93
A2	13696912	9821420	97.01	89.31	51.74
A3	10328255	7194755	96.3	88.15	51.97
B1	12050694	10872234	99.13	97.72	49.72
B2	12151044	11009081	99.16	97.77	49.59
B3	12118381	10897279	99.04	97.53	50.11
C1	12432537	10590025	99.09	97.61	50.62
C2	11875757	9302801	99.08	97.51	51.06
C3	12432537	11716106	99.18	97.84	49.72

Note: A1, A2, and A3 are fetal samples; B1, B2, and B3 are 3-month-old samples; C1, C2, and C3 are 15-month-old samples.
