# Supplementary material for: Integrated Transcriptome Analysis of miRNAs and mRNAs in the Skeletal Muscle of Wuranke Sheep
Source: Genes (Basel). 2023 Oct 31;14(11):2034. doi: 10.3390/genes14112034 (PMC10671749; doi:10.3390/genes14112034)
Supplement: Supplementary file 1 [file genes-14-02034-s001.zip › Supplementary materials/Table S4.pdf]

Table S4. Expression levels of top 10 highly expressed known miRNAs

miRNA	Fetal (ave. normalized counts)	3-month-old (ave. normalized counts)	15-month-old (ave. normalized counts)
oar-miR-133	340740.58	3002046.59	3438371.64
bta-miR-1	118670.11	1171679.50	1056407.28
oar-miR-26a	197141.95	324122.68	342909.37
oar-miR-143_R+1	57954.32	475884.82	282383.10
oar-let-7a	377362.60	178959.63	178744.08
oar-miR-127_L-1	687379.38	30951.87	14202.53
chi-miR-451-5p_R+4	32212.16	331050.01	351419.80
chi-miR-378-3p	133904.65	298905.00	243592.90
oar-miR-125b_R+1	216645.73	158314.04	134291.34
chi-miR-206_R-1	292338.20	111175.92	96857.84
