# Supplementary material for: Integrated Transcriptome Analysis of miRNAs and mRNAs in the Skeletal Muscle of Wuranke Sheep
Source: Genes (Basel). 2023 Oct 31;14(11):2034. doi: 10.3390/genes14112034 (PMC10671749; doi:10.3390/genes14112034)
Supplement: Supplementary file 1 [file genes-14-02034-s001.zip › Supplementary materials/Table S5.pdf]

Table S5. Expression levels of top 10 highly expressed novel miRNAs

miRNA	Fetal (ave. normalized counts)	3-month-old (ave. normalized counts)	15-month-old (ave. normalized counts)
PC-3p-282_33957	5067.11	4894.16	4684.81
PC-5p-1150_3588	338.46	301.38	570.13
PC-3p-2138_1335	1068.98	51.36	54.17
PC-5p-2779_892	187.75	196.17	301.31
PC-3p-3522_622	363.12	20.51	9.35
PC-5p-3703_578	32.78	141.77	145.58
PC-3p-9379_187	4.06	82.65	202.05
PC-3p-15581_117	0.00	123.39	162.93
PC-5p-10001_176	3.21	83.04	189.87
PC-3p-4027_504	123.44	73.08	75.23
