# Supplementary material for: Integrated Transcriptome Analysis of miRNAs and mRNAs in the Skeletal Muscle of Wuranke Sheep
Source: Genes (Basel). 2023 Oct 31;14(11):2034. doi: 10.3390/genes14112034 (PMC10671749; doi:10.3390/genes14112034)
Supplement: Supplementary file 1 [file genes-14-02034-s001.zip › Supplementary materials/Table S6.pdf]

Table S6. Summary of mRNA sequencing data

Sample	Raw reads	Valid reads	Mapped reads	Q20 (%)	Q30 (%)	GC content (%)
A1	12472153	9700885	48900793	99.74	95.82	50.00
A2	13696912	9821420	55623639	99.55	95.02	48.50
A3	10328255	7194755	53334762	99.61	94.95	49.00
B1	12050694	10872234	40110679	99.63	93.95	47.00
B2	12151044	11009081	40428908	99.67	93.92	47.00
B3	12118381	10897279	51414258	99.34	92.21	47.00
C1	12432537	10590025	48180297	99.48	93.14	47.00
C2	11875757	9302801	52761574	99.31	92.05	47.00
C3	12432537	11716106	45407150	99.26	91.88	48.00

Note: A1, A2, and A3 are fetal samples; B1, B2, and B3 are 3-month-old samples; C1, C2, and C3 are 15-month-old samples.
