# Supplementary material for: Integrated Transcriptome Analysis of miRNAs and mRNAs in the Skeletal Muscle of Wuranke Sheep
Source: Genes (Basel). 2023 Oct 31;14(11):2034. doi: 10.3390/genes14112034 (PMC10671749; doi:10.3390/genes14112034)
Supplement: Supplementary file 1 [file genes-14-02034-s001.zip › Supplementary materials/Table S1.pdf]

Table S1. Primer sequences of miRNAs for RT-qPCR used in this study

miRNA	Primer sequences (5′-3′)
oar-miR-1185-5p	RT: GCACTTCAGTGTCGTGGTCAGTGACGGCAATTTGAAGTGCGAACATA
	Forward: CAGACGACCATCAGAGAGGATACCCTTTGTA
	Reverse: GCACTTCAGTGTCGTGGTCAGTGACGGCAATT
oar-miR-299-5p	RT: GCACTTCAGTGTCGTGGTCAGTGACGGCAATTTGAAGTGCATGTATGT
	Forward: CAGACGACCATCAGTGGTTTACCGTCCCAC
	Reverse: GCACTTCAGTGTCGTGGTCAGTGACGGCAATT
oar-miR-370-3p_R-2	RT: GCACTTCAGTGTCGTGGTCAGTGACGGCAATTTGAAGTGCACCAGGTT
	Forward: CAGACGACCATCAGGCCTGCTGGGGTGGAA
	Reverse: GCACTTCAGTGTCGTGGTCAGTGACGGCAATT
oar-miR-380-3p	RT: GCACTTCAGTGTCGTGGTCAGTGACGGCAATTTGAAGTGCAGACGTGG
	Forward: CAGACGACCATCAGTATGTAATGTGGTCC
	Reverse: GCACTTCAGTGTCGTGGTCAGTGACGGCAATT
oar-miR-133	RT: GCACTTCAGTGTCGTGGTCAGTGACGGCAATTTGAAGTGCACAGCTGG
	Forward: CAGACGACCATCAGTTGGTCCCCTTCAACC
	Reverse: GCACTTCAGTGTCGTGGTCAGTGACGGCAATT
oar-miR-150	RT: GCACTTCAGTGTCGTGGTCAGTGACGGCAATTTGAAGTGCCACTGGTA
	Forward: CAGACGACCATCAGTCTCCCAACCCTTGTA
	Reverse: GCACTTCAGTGTCGTGGTCAGTGACGGCAATT
oar-miR-27a	RT: GCACTTCAGTGTCGTGGTCAGTGACGGCAATTTGAAGTGCGCGGAACT
	Forward: CAGACGACCATCAGTTCACAGTGGCTAAG
	Reverse: GCACTTCAGTGTCGTGGTCAGTGACGGCAATT
oar-miR-29a_R+1	RT: GCACTTCAGTGTCGTGGTCAGTGACGGCAATTTGAAGTGCTAACCGAT
	Forward: CAGACGACCATCAGTAGCACCATCTGAAAT
	Reverse: GCACTTCAGTGTCGTGGTCAGTGACGGCAATT
PC-5p-3703_578	RT: GCACTTCAGTGTCGTGGTCAGTGACGGCAATTTGAAGTGCTCCAGGGC
	Forward: CAGACGACCATCAGGATGAGGCTCAGCGAGC
	Reverse: GCACTTCAGTGTCGTGGTCAGTGACGGCAATT
U6	Forward: CTCGCTTCGGCAGCACAT
	Reverse: GAACGCTTCACGAATTTGCGT
