# Supplementary material for: Integrated Transcriptome Analysis of miRNAs and mRNAs in the Skeletal Muscle of Wuranke Sheep
Source: Genes (Basel). 2023 Oct 31;14(11):2034. doi: 10.3390/genes14112034 (PMC10671749; doi:10.3390/genes14112034)
Supplement: Supplementary file 1 [file genes-14-02034-s001.zip › Supplementary materials/Table S2.pdf]

Table S2. Primer sequences of mRNAs for RT-qPCR used in this study

mRNA	Primer sequences (5′-3′)
FBP2	Forward: GACCACAGGCACCCAACC
	Reverse: CTCATCACCAGCATTCACTCACTT
MYH10	Forward: TCATCTACAACCCTGCCACTCA
	Reverse: CGCCAACTCCACCATAACTTCAT
HDAC1	Forward: CGCCCTCACAAAGCCAATG
	Reverse: GCCACAGAGCCACCAGTAG
ITGB1	Forward: GGAGCCACAGACATTCACACTAA
	Reverse: ACGAAAGAGCCAAACCCGATT
COL4A1	Forward: AGAGCGGAGCGAGATGTTCA
	Reverse: GGAGGAGGGAGTAGCACCATTC
HSP90B1	Forward: GACACCGCAGAAGACACAGA
	Reverse: TTCACATTCCCTCTCCACACAG
CANX	Forward: TTGTCATCCTCTTCTGCTGTTCTG
	Reverse: TTCTCTTCACCTTCCTCCTCTTCA
GAPDH	Forward: GGTCGGAGTGAACGGATTTG
	Reverse: TGGCAACGATGTCCACTTTG
